# Women’s perspectives on the ethical implications of non-invasive prenatal testing: a qualitative analysis to inform health policy decisions

**DOI:** 10.1186/s12910-018-0267-4

**Published:** 2018-04-16

**Authors:** Meredith Vanstone, Alexandra Cernat, Jeff Nisker, Lisa Schwartz

**Affiliations:** 1 Department of Family Medicine, McMaster University, DBHSC 5003E, 100 Main St W, Hamilton, ON L8P 1H6 Canada; 2 Centre for Health Economics and Policy Analysis, McMaster University, Hamilton, ON Canada; 3 Life Sciences Program, McMaster University, Hamilton, ON Canada; 4 Department of Obstetrics and Gynecology, Western University, London, ON Canada; 5 Children’s Health Research Institute, London, ON Canada; 6 Department of Health Research Methods, Evidence and Impact, McMaster University, Hamilton, ON Canada

**Keywords:** Non-invasive prenatal testing, Prenatal screening, Qualitative research, Ethics, Health policy, Patient engagement

## Abstract

**Background:**

Non-Invasive Prenatal Testing (NIPT) is a technology which provides information about fetal genetic characteristics (including sex) very early in pregnancy by examining fetal DNA obtained from a sample of maternal blood. NIPT is a morally complex technology that has advanced quickly to market with a strong push from industry developers, leaving many areas of uncertainty still to be resolved, and creating a strong need for health policy that reflects women’s social and ethical values. We approach the need for ethical policy-making by studying the use of NIPT and emerging policy in the province of Ontario, Canada.

**Methods:**

Using an adapted version of constructivist grounded theory, we conducted interviews with 38 women who have had personal experiences with NIPT. We used an iterative process of data collection and analysis and a staged coding strategy to conduct a descriptive analysis of ethics issues identified implicitly and explicitly by women who have been affected by this technology.

**Results:**

The findings of this paper focus on current ethical issues for women seeking NIPT, including place in the prenatal pathway, health care provider counselling about the test, industry influence on the diffusion of NIPT, consequences of availability of test results. Other issues gain relevance in the context of future policy decisions regarding NIPT, including funding of NIPT and principles that may govern the expansion of the scope of NIPT. These findings are not an exhaustive list of all the potential ethical issues related to NIPT, but rather a representation of the issues which concern women who have personal experience with this test.

**Conclusions:**

Women who have had personal experience with NIPT have concerns and priorities which sometimes contrast dramatically with the theoretical ethics literature. These findings suggest the importance of engaging patients in ethical deliberation about morally complex technologies, and point to the need for more deliberative patient engagement work in this area.

**Electronic supplementary material:**

The online version of this article (10.1186/s12910-018-0267-4) contains supplementary material, which is available to authorized users.

## Background

This study examines women’s perspectives on the fast-growing, ethically-complex technology: non-invasive prenatal testing (NIPT). NIPT is used to screen for genetic characteristics including chromosomal aneuploidy (e.g. Trisomy 21 or Down syndrome), sex chromosome anomalies, and fetal sex, from fetal DNA obtained in a sample of maternal blood [1–3]. NIPT can be used as early as 9 or 10 weeks gestation [3–6]. It is more accurate than the tests involved in traditional forms of prenatal screening [7–10]. However, it is still a screening rather than a diagnostic test, meaning that the results should be understood to indicate risk rather than make a diagnosis. The “non-invasive” nomenclature designates that NIPT is not associated with the risk of miscarriage that exists with diagnostic tests such as amniocentesis or chronic villus sampling (CVS) [3–6]. These benefits aside, NIPT is an example of what Hoffman has called a “morally challenging technology” [11] because it poses moral issues which are broader than the specific technology, such as sex selection and eugenics, and because it has implications for larger groups than those who interact directly with the technology, such as persons with disabilities. NIPT poses some new moral issues, and it exacerbates or reinforces others which have long been identified as related to other forms of prenatal testing. New ethical issues posed by NIPT include the worry that it may facilitate sex selective termination by providing accurate information about fetal sex within the first trimester [5, 12]; the availability of potentially anxiety-provoking information about genetic traits of unknown clinical significance [13, 14]; and the scientific possibility of using NIPT for an expanded slate of conditions that have decreasing impact on functioning, or which are late-onset conditions, or conditions with variable penetrance [15]. The development and diffusion of NIPT has been driven by industry which has resulted in the test being market before validated, unbiased evidence of efficacy was available, and which may restrict future development and affordability of the test [14–16]. In countries where NIPT is not publicly funded, there may be equity issues surrounding who can access NIPT and what other opportunities that access creates (i.e. earlier access to invasive testing and termination) [5, 13, 17]. Exacerbated or continuing ethical issues include the concern that increasing levels of routinization and diminished facilitators for women to make informed choices may result in increased discrimination against people living with disabilities [18–21].

NIPT has diffused quickly into mainstream prenatal practice in many high resource countries, largely driven by industry imperatives [22]. In Ontario, Canada, where this study takes place, NIPT has been commercially available since 2011, and publicly funded for women at high risk of aneuploidy since 2014 [23, 24]. Ontario offers publicly funded prenatal screening and diagnosis as part of a provincial health insurance plan that funds hospital and physician services. All pregnant women are offered prenatal screening through a provincial program established in the mid 1990s [25]. In 2014, NIPT was publicly funded on a case-by-case basis as a form of second-tier screening, most commonly accessed when initial screening yields results indicating the pregnancy to be at higher risk. Depending on the initial screening modality chosen, the initial screening results may not be available until the middle of the second trimester, so under the current policy, many Ontario women consider the offer of publicly funded NIPT quite late in pregnancy [24]. Other Ontario women may access publicly funded NIPT if they are over the age of 40 at the expected date of delivery, or have previously had an affected pregnancy. NIPT is available in Ontario as a private pay technology at any time.

As a result of the quick diffusion of NIPT, policy makers in many jurisdictions are tasked with making a number of decisions regarding how best to implement NIPT in the prenatal care pathway, which health professionals should be involved in counselling, and how to handle the growing number of clinical applications for NIPT [26]. While some jurisdictions have started to publicly fund NIPT in specific circumstances [24, 27–29], many other jurisdictions are in the process of assessing this technology prior to policy decision-making, through Health Technology Assessment, or other policy-making processes.

The importance of including patient and user perspectives has been well studied within the policy context of health technology assessment (HTA) [30–32]. Enthusiasm for patient engagement is not limited to HTA; other health policy venues are increasing their capacity and refining their approach to including patient and public perspectives in policy decisions e.g [33, 34]. Patient perspectives provide an important source of information for ethical policy-making, particularly by illuminating issues which may not be considered or may be de-prioritized when decision-makers focus on clinical efficacy and cost [31, 35–37]. The growing number of women who have had personal experience with NIPT can contribute personal values, experiences, and beliefs to inform future policy decision-making about this technology. The perspectives of users and other stakeholders can provide unique understandings of policy implications. Critical reflection on these perspectives may provide insight into the ethical implementation of NIPT, contributing to policy that is comprehensive, morally justifiable, and able to gain public support [30, 32, 35]. Incorporating patient perspectives also promotes policy that is responsive to the needs and wishes of the people who will be effected by the policy decisions, and in so doing increases the likelihood that the policy will be accepted and adopted [30, 35, 38].

Since the concept of NIPT was first introduced, ethical analysis of this technology has proliferated, primarily representing theoretical perspectives, e.g. [15, 39–43]. As the uptake of NIPT has grown, empirical research on the views, opinions, and knowledge of the public, pregnant women, and clinicians has also occurred [24, 44–50]. Despite these two burgeoning research streams, there has been little cross-over, with only a few of the empirical examinations of stakeholder experiences explicitly addressing the ethical implications of their findings [26, 51–54]. Without a strong connection between theory and empirical evidence of stakeholder opinions towards NIPT, we lack a rigorous understanding of what ethical issues matter to the users of this technology. Towards the goal of contributing to ethical policy that reflects patient preferences, we examined women’s perceptions of ethics issues related to NIPT by asking “How do women understand the ethics implications of the implementation of NIPT in Ontario, Canada?” These findings may inform policy deliberation in a wide variety of ways, and their usefulness will vary according to the specific policy question under consideration and the mode of policy decision-making. These findings may contribute to construction of patient-relevant outcomes in an economic analysis [55], determination of optimal placement of NIPT in the clinical pathway, design of patient education materials, or inform ethical and social analysis in a health technology assessment of NIPT.

## Methods

The methods for this adapted constructivist grounded theory study have been described in detail elsewhere [24]. Briefly, we conducted thematic analysis on semi-structured interview data collected within the context of a constructivist grounded theory study to describe women’s perceptions of ethical issues pertaining to NIPT. We conducted semi-structured interviews with 38 women purposively sampled because they had a variety of personal experiences with NIPT (Table 1). All women lived in Ontario, but some had accessed NIPT in other jurisdictions. To ensure our sample included women with diverse experiences, participants were recruited through a high risk prenatal diagnosis clinic using a consent-to-contact form presented by their clinician, ads on online classified sites (e.g. Kijiji), online pregnancy and parenting sites, and via snowball sampling. We initially recruited women living in Ontario who had any personal experience with NIPT. As the project progressed, we used the iteration between data collection and analysis to theoretically sample particular groups of interest (e.g. women who declined NIPT, women who were offered NIPT in the preconception period). We recruited these groups by tailoring our strategy in several ways, e.g. asking clinicians at the clinic to give the consent-to-contact form to certain types of women, rather than any woman offered NIPT, and by screening interested women who contacted us from our online advertisements.

**Table 1:** Testing Pathway of Participants (*N* = 38)

	Number	Percent
Number of Women	38	
Number of Offers of NIPT^a^	42	
Accepted NIPT at Least Once		
Yes	30	79%
No	8	21%
Funding for NIPT		
Eligible for public funding, accepted NIPT	19	45%
Eligible for public funding, declined NIPT	7	17%
Ineligible for public funding, accepted NIPT	13	31%
Ineligible for public funding, declined NIPT	3	7%
Point in Pregnancy NIPT Considered		
Preconception	6	14%
First Trimester	12	29%
Second Trimester	24	57%
Reason for Considering NIPT		
Advanced Maternal Age (≥ 40 years)	2	5%
Previous Affected Pregnancy	3	7%
Recurrent Unexplained Miscarriages	1	2%
Screened positive from FTS	4	10%
Screened positive from IPS	14	33%
Soft markers on 2nd Trimester Anatomy Scan	8	19%
No elevated risk factors, desire for general information	10	24%

*FTS* First Trimester Screening, *IPS* Integrated Prenatal Screening

^a^35 women discussed one offer of NIPT. Two women discussed two separate instances in which they were offered NIPT, one woman discussed three separate offers of NIPT

We explained our study to participants as aiming to gather women’s values, experiences, and opinions on NIPT in the hopes of potentially informing future policy about this new health technology. After eliciting personal experiences and values about this technology (findings described in [24]), we entered an in-depth discussion of ethical implications by asking women to describe their thoughts on the advantages, disadvantages, concerns and benefits of this new technology. As interviewers, we adapted our conversational style to match the level of engagement each participant had with these ethics ideas. Some women needed little prompting, and spoke at length about their concerns and excitement about NIPT, in response to broad questions designed to allow unanticipated ethical issues to emerge. Others required a more structured approach, and we used interview probes to explore thoughts related to regulation and funding, expansion to other conditions, implications for reproductive decision-making and social impacts. The interview guide is provided as a supplementary file to this manuscript (see Additional file 1) While the women in this study were familiar with NIPT by virtue of their own involvement with it, we did not provide any education about the technology and asked women to respond to our questions based on their existing knowledge, opinions and beliefs.

Two researchers (MV, AC) conducted a thematic analysis of ethics implications identified by women. We analyzed our data using the staged coding technique of grounded theory, looking specifically for mentions of issues which we judged to have ethical implications. Participants did not need to explicitly identify an issue as ethical for it to be included in our coding; we also identified implicit ethical issues. For example, participants often discussed the price of NIPT as a barrier to accessing the test. While discussions centered on the financial implications, we would code this type of talk as ethically relevant because it is related to issues such as equity of access. Our identification of ethics issues was informed by a familiarity with the broad literature on ethical implications of prenatal screening. We did not use any particular ethics theory as a theoretical lens for this descriptive analysis, but as reflexive researchers we recognize that our existing ethical commitment to theories such as relational autonomy and feminist ethics [56, 57] colour the way we identify and describe ethical issues.

We began by working independently to conduct line-by-line coding with the intent of identifying ethical issues. We met periodically throughout this process to discuss and compare the issues we were identifying. After line-by-line coding was completed, we consulted with the rest of the research team to identify a direct for focused coding. Further rounds of focused coding permitted the condensation of these themes into categories. Discussion with the broader team helped form the findings presented in this manuscript. NVivo® 10 software was used for data management.

This study received research ethics approval from the Hamilton Integrated Research Ethics Board.

## Results

Thirty-eight women participated. Table 1 describes the range of experiences these women had with NIPT and Table 2 describes their demographics. In general, our participants were older, more educated and more likely to receive care from a family physician than the average Canadian pregnant woman [58, 59]. Our sample reflects a diverse array of experiences with NIPT, including women who accepted and declined the test, and those who were offered NIPT at all stages of pregnancy and for a variety of indications.

**Table 2:** Demographic characteristics of study participants (*N* = 38)

	Number	Percent
Pregnancy		
Singleton	37	97%
Twin	1	3%
Primary prenatal care provider		
Family physician	17	45%
Obstetrician	13	34%
Midwife	2	5%
Fertility specialist	2	5%
Family physician + obstetrician	3	8%
Family physician + midwife	1	3%
Age at delivery (mean = 35.4 yrs)		
25-29 years	2	5%
30-34	14	37%
34-39	16	42%
40+	6	16%
Number of existing children		
Zero	9	24%
One	19	50%
Two	9	24%
Three or more	1	3%
Location of Residence		
Urban	26	68%
Suburban/town	9	24%
Rural	3	8%
Religiosity		
Considers self “religious”	13	34%
Christian	7	18%
Jewish	5	13%
Christian + Jewish	1	3%
Does not consider self “religious”	25	66%
Highest level of education		
High school degree	1	3%
College degree	7	18%
University undergraduate degree	13	34%
University graduate or professional degree	17	45%

We identified many ethical issues related to the research participants’ experiences with the process of NIPT. Many of these issues (e.g. routinization, impact on people with disabilities) have been well described in existing literature about other prenatal tests. We concentrate here on describing the identified ethics issues unique to, or exacerbated by, NIPT and have organized these results based on their primary level of impact. Those which pertain to women currently seeking the test are presented first, followed by issues which gain relevance in the context of future policy decisions. Four categories of ethical issues are identified: Inconsistent Implementation of a Quickly Evolving Industry-Driven Technology, Early Availability of Results, Financial Considerations, and Potential for Expansion of NIPT.

We do not claim that the issues identified by the women in our study represent an exhaustive list of possible ethical implications of NIPT. Rather, these findings represent the understandings, concerns, and priorities of women who have had personal experience with this technology in Ontario, Canada.

Quotes from participants are contextualized with information about whether that woman accepted (A) or declined (D) NIPT, the trimester of pregnancy in which NIPT was offered (1, 2 or P for preconception). The “$” notation indicates the woman was eligible for publicly funded NIPT. Accordingly, D1$ indicates a woman who declined a first-trimester offer of publicly funded NIPT. Three women were offered NIPT more than once, and each offer is noted individually. For example, “A2 A1 A1” describes a woman who paid privately for NIPT in multiple pregnancies.

### Inconsistent implementation of a quickly evolving industry-driven technology

Participating women identified a number of concerns about the way that NIPT has been implemented in Ontario, where industry imperatives have meant wide dissemination of the technology in absence of systematic regulation or organization at a policy level. As a result of this organic dissemination, women expressed dissatisfaction with several aspects of NIPT implementation including inconsistency in the information and counselling received from different health care providers and the restriction of the offer of NIPT to women who are at identifiably high risk. We implicitly identified several other issues related to industry influence on dissemination.

Twenty women in our study discussed an unsatisfactory experience with the way health care providers discussed NIPT. Women who discussed it with their primary care provider typically remarked that they did not perceive this person to be well-informed about the test: “*I talked to my doctor about it and I’m not sure she knew too much about what the risks are. It didn’t ease my mind very much*” (A2$). In contrast, those who had the opportunity to discuss NIPT with a specialist provider were generally pleased with the interaction: “*The genetic counsellor was amazing so whatever their background is, I don’t know but they’re doing a good job. Not to discredit my family physician but I didn’t feel like they were very prepared or good at explaining it to me*” (A2$).

We identified an inconsistency between the way that NIPT is currently offered in Ontario and women’s preferences for who should offer NIPT and when that offer should occur. Few of our participants were offered NIPT by a primary care provider in absence of a particular risk factor, but many women favour this approach: “*It needs to start with the GPs. I mean, that’s the first person typically that you’re going to see when you find out you’re pregnant and I think it needs to be a part of the conversation right from the beginning*.” (A2$) Our participants were overwhelmingly in favour of all women being offered NIPT as part of the initial conversation on prenatal screening, regardless of whether or not NIPT is publicly funded. This view remained consistent even when opinions were probed in response to the cost burden of NIPT for women who did not qualify for public funding, the time pressures on primary health care providers, the potential for iatrogenic anxiety, and the for-profit nature of this test: “*When you go in for that first appointment and the doctor lays out what all the testing is, I don’t think it would be such a big, scary deal if it was just made part of that. An option within routine care. I don’t know why somebody would withhold the possibility of having that test*” (AP).

Across our dataset, we identified several issues of inconsistent implementation related to the private development and diffusion of NIPT through industry, without governmental regulation. These ethics issues were mostly identified implicitly, and include the competition between brands of NIPT, a concern that physicians or hospitals were incentivized to encourage uptake of NIPT, and strong marketing messages about NIPT. Regarding competition between brands, we heard very little about making a choice between the available NIPT brands. Typically, those who discussed multiple brands focused on logistical reasons for their choice: “*I had the Panorama instead of the Harmony just because that’s what’s offered at the lab*.” (A2$). Most women were not concerned about the relationship between this private-pay test and their health care provider, with only three women raising the issue. For these women, the perception that a physician or hospital may benefit from the uptake of a prenatal screening test may disrupt the trust in the physician-patient relationship: “*I would say he was pushing during that appointment. … He had pamphlets in his office that he was distributing. I don’t know if a rep had been into the office and if they were getting any kind of a cut*.” (D2$). We saw evidence that strong marketing messages about NIPT were internalized by many participants, leading to inaccurate understandings of the test. This phenomenon was most notable around women’s understanding of accuracy rates. Most women conflated the detection rate for Trisomy 21 to be the detection rate for all conditions. Trisomy 13 and 18 have a lower detection rate and positive predictive value, or the probability that a woman with an affected pregnancy would receive a positive result, especially in normal-risk women: *“It’s so precise. Like 99% accurate. The accuracy rate is amazing. I think that’s what’s great about it.”* (A2$) Very few participants were skeptical about detection and accuracy rates, with only a small number of highly educated women mentioning this issue: “*it’s a newer test, so what is the error rate*?” (D2$).

### Early availability of results

NIPT can be used as early as 9 or 10 weeks gestation, depending on the brand selected, with results available approximately 10 days later. This is significantly earlier than results from other screening tests which may not be available until 14-20 weeks, depending on the testing modality. The early availability of NIPT results was mentioned often, and we identified two related ethics issues. First, because results are available within the time frame that pregnancy termination is readily available, some women feared that NIPT would be used for sex selection. “*If it can tell you gender that early, well then you have a lot of options in terms of terminating if it’s not the gender you wanted. I’d be very wary of that, if you are using it for that purpose*” (A2$). While the topic of sex selection dominated this conversation, many participants mentioned a fear that the conditions detected by NIPT would expand, and it may be used to terminate for reasons they judged distasteful. “*You get into a sticky situation because then you have people who will abort for what some of us consider irresponsible reasons*” (DP). The applications of NIPT that women judged distasteful varied by participant and will be discussed further at the end of this section.

While the possibility of early termination was seen as negative in some circumstances, it was seen as positive in other situations, because an early termination would obviate some of the physical and emotional difficulties encountered with later termination: “*You feel the baby moving and the emotional impact of it—it’s already a terrible decision to make but the longer you stay pregnant the worse it is.*” (D1$).

All participants supported earlier access to NIPT, and access to NIPT for all women. This opinion was consistent even among those who declined NIPT, those who stated they would never consider terminating their own pregnancy, and even after the interviewer probed about the possibility of using NIPT for sex selection. This sentiment was so strong that we could identify an argument that patient-centered policy in this area should enable rather than restrict access to a technology which would facilitate earlier decisions about termination. “*I would be outraged if it existed and I wasn’t given the opportunity*” (A2 A1 A1). This sentiment was particularly strong amongst women who understood NIPT to be a superior substitute for existing prenatal screening pathways, and expressed their willingness to substitute NIPT for current screening methods: “*If this screening tool is available, and if it’s that much better than what’s currently offered, I don’t know why they wouldn’t substitute it. Just like if a better medical practice comes along, then we adopt that practice*” (A1$).

### Financial considerations

Some research participants were concerned with the cost implications of NIPT to the publicly-funded healthcare system. “*It’s always hard to know how to distribute the resources*” (D2$) Many women mused about the cost differences of NIPT versus the existing prenatal screening pathway, on the budget impact of publicly funding NIPT, and on the cost of the additional counselling and health care providers that would be required: “*The other screening that I did involved two blood tests and an ultrasound so I can’t imagine that’s cheap either*” (A2$).

Within women’s discussions of whether NIPT should be publicly funded and if so, for whom, we identified several ethical issues related to the economic assessment of this technology. For example, what outcomes should be used in an assessment of cost-effectiveness? Many women suggested that the government might consider potential savings in long-term health care costs when affected pregnancies are terminated: “*What they’re paying out in testing they might be saving down the line in cost of care for highly disabled children, their hospital care, their intensive care, whether they make it beyond a certain number of weeks, months, whatever*” (D1$). Others cast this issue as a need to plan for the support of people with disabilities, rather than calculating how much money would be saved by preventing those births: “*They* [parents] *will take care of their kids* [with disabilities] *but they do need the support from society and then we can actually plan long term resources*” (A2$).

A few women suggested the need to consider non-monetary outcomes such as reducing the number of miscarriages associated with invasive testing. “*I feel for women who perhaps have issues and really want to find out but don’t want to run the risk of a miscarriage*” (D1$). Reducing stress, worry, and offering reassurance was another common non-monetary outcome of NIPT. “*I think it should be paid for the moment the obstetrician hands over the pamphlet to a pregnant woman and says there is a chance that your fetus could have this. Because, it is at that moment that incredible stress is being put upon a pregnant woman and it’s not good for anyone. It’s not good for the family unit*.” (A2).

Within these conversations, women often identified how funding NIPT is related to, or in tension with, what they identified to be Canadian societal values about universal health care. Some saw public funding of NIPT only for women at high risk of aneuploidy as in conflict with our national commitment to universal care: “*I think in our Canadian liberal equal opportunity system if it’s going to be covered it should be covered* [for everyone]*.*” (AP) Others recognized that a publicly funded healthcare system is always required to make tough decisions to allocate resources, and that stratifying access by risk is a reasonable way to do this: “*If you look at our healthcare system, you want it to be appropriate and you want it based on needs and not on desire*.” (D1$) When describing the current arrangement of NIPT, many women recognized that the existing implementation of the technology might be described as a “two tier” system, where inequity of access exists based on ability to pay: “*If you can pay, then you pay. But then that’s a two-tiered medical system and nobody likes that*.” (A2$) Many participants recognized the inequity created by the need to pay privately system, and the difficulty faced by women who would struggle to pay the cost of NIPT: “*just because we could find $800 doesn’t mean that we deserve to know that our baby was okay more than anyone else does.*” (A2).

### Potential for expansion of NIPT

Our results so far have focused on the ways that NIPT is currently used and understood by women. However, our discussions often branched into more speculative waters as women talked about future potential uses or consequences of NIPT. In these conversations we identified a tension between women’s desire for reproductive autonomy—to gain information about their pregnancies and make decisions about their pregnancy based on that information, their values, and personal circumstances—and a sense of unease or discomfort with how other women might do the same. While many of our participants wished for some sort of regulation about the potential uses of NIPT, they were uncomfortable with defining boundaries or specifics of what this regulation would entail.

Almost all participants were able to identify a scenario where NIPT could be used for something they felt should be restricted or prohibited through regulatory means. Participants had widely varying thresholds for restricting the use of NIPT. Some common suggestions for prohibited uses of NIPT included sex selection, termination on the basis of conditions that had limited, localized impairments (e.g. deafness or blindness), termination on the basis of adult-onset conditions (e.g. Huntington’s disease), termination on the basis of physical traits that do not impair functioning (e.g. attached ear lobes). Many women made the distinction that their objection was not to obtaining knowledge from NIPT, but to the idea that someone might use that knowledge to terminate their pregnancy:

“*The deaf and blind communities would argue that they are not disabilities they are just their way of life. … if someone said to me, we can tell you whether the baby is going to be deaf or not, do you want to know? I would probably say yeah I want to know*. [but I wouldn’t choose to terminate for that] … *Maybe someone else would terminate, maybe, that is their choice. I don’t think people should be allowed to tell other people how to procreate, or reproduce*” (AP)

Others felt that “frivolous” applications should not be offered in testing at all, regardless of who is paying or what is done with the information:

*“To be honest, I think that my answer is the same whether we’re paying or the government is paying. I think that it’s helpful to know anything that can affect the birth of a healthy child or a childhood disease they can develop like cystic fibrosis and such but ... I think it’s crossing over to a whole new realm when you start talking about finding ... whether they’re going to have whatever colour hair or things that are not exactly relevant for when the baby is still in the womb as the baby and the child. I don’t know, I think that there should be some limits.”* (A2$)

On the other hand, most women also expressed a strong belief in bodily autonomy and control, access to information about her pregnancy, and the choice to do what she wanted with that information: “*I will make that decision. This is my baby not your baby so if there are options out there, I should know about them. If there’s additional tests out there that are available, I should know about them*” (A2$). The question of what NIPT should be used to test for, and who should decide or control this process aroused discussions of choice, value systems, autonomy, and the cumulative impact of individual decisions on the diversity of society. “*I don’t like the government making choices for me, but who am I to say where they stop*” (D2$).

Through the discussions of several women we were able to identify a discomfort with the idea of one policy or regulation that would apply to all women, or all circumstances: “*It’s tough because we’re dealing with such a broad population of people with very different moral standards and ethical codes*.” (D1) From most of our participants we heard an acceptance of ambiguity, and a reluctance to enforce rules or regulations about reproduction, even as they recognized the necessity of these policies. “*I don’t know how you make that call, I don’t know the answer to that. … That’s such a personal thing and I would never want to make that decision for somebody else, but you have to have policies that affect everybody, too, so I don’t know.”* (D2$).

## Discussion

In this paper, we describe some of the ethical concerns of women who have personal experience with NIPT, contributing patient perspectives and experiences to policy considerations of this new technology. Our participants highlighted a variety of ethics issues, many of which are related to access and self-determination; they were clear in their message that each woman should be able to choose when and how she will engage with NIPT, and how she will use that information. The women in our study strongly supported public funding for some, but not all applications of NIPT. They were comfortable with the general idea of governmental regulation of this technology, but were reticent to suggest particular limits on how NIPT should be used. There was little consensus on the limits that women did suggest, marking an important area for future research and patient engagement. Within the priorities expressed by our participants, we note both agreement with and divergence from some of the normative theoretical positions identified in the existing ethics literature, and we identify these agreements and disagreements throughout this section.

### Ethical issues related to inconsistent implementation of NIPT

Participants identified several ethical issues resulting from the way NIPT has been implemented, both the ad-hoc industry-driven implementation and the 2014 Ontario policy requiring a case-by-case demonstration of risk to access publicly funded NIPT. We identified a theme across these issues pertaining to the facilitation or restriction of women’s autonomous choices about how they wish to engage with NIPT. Some implementation issues related to a perception of potentially undue pressure to test, such as the concerns a small number of women mentioned about industry influence on advertising and informational materials, incentivized health care professionals, and competition between brands. These concerns are consistent with ethics issues identified in the theoretical literature [16, 60, 61], although they were only mentioned by a small number of very educated women in our sample. The dominant view across our participants was a concern that the way NIPT has been implemented restricts the ability of individual women to make an autonomous choice about how they wish to engage with NIPT. This includes disappointment with incomplete and inconsistent information provided by health care professionals, but it mainly pertains to the lack of a universal offer of NIPT as early as possible in pregnancy. The expansion of the offer of NIPT, regardless of risk status or funding structure, was a primary priority of our participants.

This priority remained even when we probed around ethical arguments against expanding the offer (e.g. inequity of access, potential for iatrogenic anxiety etc.). While they recognized and affirmed the challenges we probed with, the vast majority were more concerned when they found out that NIPT was available but it hadn’t been offered to them earlier. This finding may reflect both our sampling strategy and the way NIPT has diffused in Ontario; many of our participants only learned of the existence of this test after receiving a positive screen on an earlier test. We have previously reported patients’ dismay at the use of NIPT in this prenatal care pathway [24].

Our participants’ enthusiasm for an expanded offer of NIPT contrasts with existing ethics literature detailing the numerous reasons why an expanded offer may not be prudent. One caution widely present in the literature is that an expanded offer of NIPT to all women challenges the ideal of informed decision-making because of persistent misunderstandings of accuracy, a lack of time for counselling, sparse availability of counselling specialists, and the potential that the procedurally-simple test could be easily routinized [15, 41, 42, 48, 51, 62–65]. Additional arguments in the literature against expanding the offer of NIPT to all women include: the evidence base in normal-risk women was established only recently, the private-pay nature of the test may discriminate in access, quality assurance measures are not widely in place, and primary prenatal care providers may not have the time or expertise available to offer NIPT more widely [15, 41, 42, 48, 62–64]. Most of these arguments can be summarized as “*we’re not yet ready*” rather than “*we shouldn’t*”. The strong desire of our participants to choose whether or not they wish to access NIPT lends urgency to resolving the challenges around expanding the offer of this test. This stance is supported by recent professional guidelines, such as the recommendation from the American College of Medical Genetics and Genomics that information about NIPT be provided to all pregnant women, and that various healthcare stakeholders work to ensure this technology is “accessible” to all women [66].

### Early availability of results

Women recognized both positive and negative implications of the early availability of NIPT results in pregnancy. Positively, they described how receiving NIPT results early could facilitate self-determination by providing more time to deliberate about the course of the pregnancy in the event of a finding of aneuploidy or other condition. If a woman chooses to terminate the pregnancy, our participants described several reasons why this choice is easier earlier in pregnancy. The possibility of early termination has been recognized by women in many studies as desirable because it is socially, emotionally, physically and psychologically easier, widening rather than restricting the choices a woman feels able to consider [63, 67–69].

Our participants identified many potentially negative aspects to early results which enable easier pregnancy termination. Primarily, our participants were concerned that other women may use the information from NIPT to terminate for “what some of us consider irresponsible reasons”. Many women talked about sex selection as an unethical application of NIPT. As our conversations broadened to the potential expansion of the conditions tested for by NIPT, the concern that women may use NIPT to terminate for reasons the participant deemed “frivolous” deepened. This tension between a desire for self-determination and a compulsion to regulate the self-determination of others is further discussed in the section on the potential for expanding the conditions offered by NIPT.

### Financial considerations

When discussing ethical issues related to finances, cost, and access, many women in our study leaned heavily on what they described as Canadian values for publicly-funded healthcare. We heard familiar rhetoric of scarce health care resources and the need to allocate those resources in a fair and equitable way that serves the common interest of society rather than the desires of individuals. Many women offered arguments related to resource allocation as the only justification for restricting the offer of NIPT, describing that the money spent on offering NIPT to all women may be better used elsewhere in the healthcare system, such as for the support of people with disabilities and their families. Others discussed the existing inequity of a “two-tiered” health care system where people with private means had access to NIPT which opened earlier access to other related technologies (e.g. invasive testing, termination). For these women, fairness and equity were important justifications for funding NIPT for all women.

A concern with equity of access and the potential implications of increasing public investment in a universal prenatal screening program for disability is an issue which has been raised by women in a small number of other studies [68, 70]. For example, van Schendel and colleagues describe how parents of children with Down syndrome neatly delineated the tension in this argument [68]. If NIPT is not publicly funded and only accessed by those with financial means, “double stigmatization” may occur when children with Down syndrome are more frequently born to those of lower socioeconomic status. If NIPT is publicly funded, this may increase perceived legitimacy and pressure to test, thereby increasing uptake and diverting resources (social and financial) away from the support of people with disabilities and their families [68]. The concern that increased use of NIPT will mean decreased support and possibilities for people living with disability has been well documented as a concern of women [49, 53, 68, 69, 71, 72] and ethics scholars [17, 26].

In terms of the transferability of these findings to other national contexts, most public health care systems are required to make decisions about how to allocate scarce resources in terms of the services and tests that they offer. As more genetic tests become available, the challenge of fairly allocating scarce public resources becomes an acute problem, requiring ethical reflection [73]. The views described by women in this study are relevant to the ethical challenge of priority-setting within a health system. Our participants recognized that a universal offer of NIPT would be very costly, drawing resources away from other parts of the system. For many, this was an acceptable reason to forego access to a technology they strongly desired, or to use personal resources to pay for that technology. It was also an acceptable justification for limiting access to NIPT by risk status, as a way of triaging resource use.

### Potential for expanding the conditions offered by NIPT

Throughout several categories of our findings, we noticed a tension between women’s desire for the opportunity to use NIPT the way they want (self-determination) and a desire for regulation which would restrict uses of NIPT they judged to be undesirable (e.g. sex selection). This tension between self-determination and regulation was particularly acute when we discussed future uses of NIPT, such as the use of NIPT to terminate for conditions not widely clinically available, but scientifically possible (e.g. achondroplasia, deafness) [74, 75]. This tension in our data highlights one of the challenges of using the argument of reproductive autonomy to justify prenatal screening. Promoting women’s ability to make informed decisions about which pregnancies to continue (reproductive autonomy) is often used to justify support for prenatal screening even though it may result in harm to people with disabilities and the diversity of our society. For those who hold this view, it is difficult to restrict women’s informed decision-making by delineating types of information they may or may not use to make that decision [14].

As we probed this issue with participants, many discussed the funding source for NIPT as a way of mitigating the tension between expanding and restricting the use of NIPT. Women suggested that perhaps the public health care system shouldn’t restrict certain applications of NIPT (e.g. testing in absence of identified risk, testing for conditions the participant believed to be frivolous), but neither should the public system financially support these applications. The willingness of some of our participants to allow a “free market” approach to NIPT is in stark contrast with ethical writing on the topic, where ethicists worry about the “trivialization of abortion” that may arise when NIPT is used to screen and terminate for non-medical traits under the justification of reproductive choice [76–78]. The view of many of our participants represents a “pure autonomy” or “pure choice” model, wherein reproductive autonomy and informed choice is used to justify any prenatal screening decision a woman wishes to make. The “pure choice” model has been critiqued by a number of ethicists [79, 80], but a preference for letting women and their partners choose what to test for has been expressed by participants in other empirical studies [47, 68, 69].

These findings suggest that the expansion of NIPT to conditions beyond those included in traditional prenatal screening tests is an area which requires urgent ethical and policy attention. Our participants had strong, but highly variable opinions on this issue which were often at odds with ethical scholarship on the topic. Similar studies of women’s views on the expansion of NIPT have also demonstrated a high degree of variability of opinion [68, 69]. Incorporating patient or citizen perspectives into health policy decision-making is increasingly encouraged in many national and policy contexts, but there is little guidance available on how to reconcile conflicts between the views and priorities of patients and other sources of information (e.g. professional practice guidelines, ethicists, economists, epidemiologists). It may be tempting to dismiss patient perspectives when these conflicts arise. For instance, we may point out that the women in our study are representative only of themselves, and do not include many important voices or perspectives which may affirm other forms of evidence, or offer another alternative. Or we may emphasize that the women in our study are speaking about their personal experience and values, and when presented with more information, these views may change. However, to dismiss the views of patients because they are not fully informed or because they represent only themselves demeans the principle of inclusion of patient and citizen perspectives. It is important to include these perspectives because they bring an alternative form of information: lived personal experience. To those grappling with discrepancies or conflicts between patient and expert views on health policy topics, we suggest that deliberative patient engagement approaches may be a step towards clarification of the values which inform the issue. Deliberative patient engagement approaches to research with women and citizens may help to 1) elicit nuance in these views; 2) help each side of the issue understand the issues raised by the other side, potentially identifying issues of congruence and 3) identify social values which may assist policy-makers to construct ethical and socially acceptable policy that will keep pace with scientific advances in this area [37, 81, 82]. Disagreements or discrepancies such as those we highlight here may never be agreed upon, but deliberative patient engagement approaches may be one approach to clarifying the underlying social and ethical values which guide perspectives in diverging directions, offering the opportunity for principled disagreement when one option must be prioritized over another.

### Limitations

Our ethics analysis represents the thoughts, opinions, and beliefs of women with personal experience of NIPT, as elicited through qualitative interviews. These women were older, more educated, and more likely to live in an urban area than the average Canadian pregnant woman. Their views are shaped by their individual demographics and experiences and by their socio-historical location, and are not intended to be representative of any particular population. The ethical issues identified by women with personal experience with NIPT may shift, particularly as the scientific, policy, and industry landscape that shapes the delivery of NIPT changes. As with many qualitative research studies which rely on volunteer participants, those who were willing to give their time to discuss their experiences may have a particular interest in the topic, or in research, which motivated their participation. We succeeded in including participants with a wide array of experiences of NIPT, but our group is homogeneous in other ways and so our analysis should not be considered to represent an exhaustive list of potential ethics implications of NIPT. Indeed, several issues mentioned in the literature (e.g. genetic privacy of other relations) were not mentioned by participants. This study is not patient engagement, but a research study which solicits perspectives of individuals with relevant experience of a technology. Since our participants all had personal experience with the technology, we did not provide formal education or facilitate a guided deliberation about the technology, as is common in other methods of patient engagement. Instead, we used a guided interview strategy to elicit thoughts and opinions on particular areas of theoretical interest, informed by the literature and earlier data collection. Data was analyzed through the interpretive lens of the researchers and the findings are provided for consideration by policy-makers, clinicians, and users of NIPT.

## Conclusion

This analysis surfaces ethics issues related to non-invasive prenatal screening for the purpose of policy decision-making. A wide variety of ethics issues are illuminated by talking to people with personal experiences of the health technology under study—issues that are often distinct from the issues discussed in the theoretical literature. The women in our study discussed an extensive variety of ethics implications of NIPT including some of the ethical challenges that have plagued prenatal testing for decades (e.g. facilitating informed decision-making, judgments about disability) and some new ethical issues specific to NIPT (e.g. expansion of funding and conditions, implications of early availability of information about fetal sex). However, this technique is not sufficient to elicit an exhaustive list of potential ethical issues, nor does it provide definitive normative guidance for policy decision-making. However, considering the issues identified by women as important to their experience of NIPT is a key aspect of policy-making about new technology, and when interpreted and considered through a normative lens of societal values and ethics priorities contributes to health policy development in this area.

## Additional file

Additional file 1: Interview guide used to collect data. Interview guide used to collect interview data from women. (DOCX 22 kb)

## Abbreviations

NIPT: Non-Invasive Prenatal Testing
