# Supplementary material for: Women’s perspectives on the ethical implications of non-invasive prenatal testing: a qualitative analysis to inform health policy decisions
Source: BMC Med Ethics. 2018 Apr 16;19:27. doi: 10.1186/s12910-018-0267-4 (PMC5902938; doi:10.1186/s12910-018-0267-4)
Supplement: Supplementary file 1 — Interview guide used to collect data. Interview guide used to collect interview data from women. (DOCX 22 kb) [file 12910_2018_267_MOESM1_ESM.docx]

**INTERVIEW GUIDE (WOMEN)**

**{**In preliminary conversation determine what the individual woman calls NIPT and use that term in the interview guide instead of NIPT}

1. To start, could you tell me a bit about the pregnancy you were offered NIPT for?

- Was it your first pregnancy?

- Did you have any other types of testing (now or in the past)

- What kind of prenatal care provider did you have (e.g. FP, Midwife, OB)

1. Please tell me how you first heard about NIPT.
   1. {if applicable} Did you remember this when you became pregnant, or did you hear about it again from somewhere else?
      1. If they heard about it from somewhere else, follow this- what context did you hear about it? What did you think about it then? Did you look into it further? Was it appealing? Did your thoughts on it change after being introduced to it from your doctor?
   2. What point in your pregnancy were you? How had the pregnancy gone up until that point?
2. When did you first learn about the possibility of more testing?
   1. How did you feel about that?
   2. Did learning about NIPT change this feeling?
3. How was NIPT explained to you?
   1. Did they give you any information about what it can test for? Were you familiar with these conditions? Did you know someone with this?
   2. Anything about how is the test done?
   3. Did they explain to you how long it would take to get results?
   4. What about if the results come back to say that your baby is affected. Did they talk about what happens then? (confirmatory amniocentesis, timing)
   5. Did they mention the different companies that offer NIPT?
4. So, I am hearing from you that [reiterate statements, e.g. the GCs did a great job]. Glad to hear that. I’m wondering what information they shared was the most confusing, what you had questions about or wanted more information on.
5. Were you offered or aware of any other types of prenatal testing?
   1. Have you done any of these types of tests in the past? (past pregnancies?)
   2. Prompt with IPS, amnio, CVS if necessary
   3. How did NIPT compare with those other tests?
   4. Did you understand the differences between NIPT and the other tests available, like IPS or amniocentesis? {important} What information was available to you about this?
   5. What were the appealing features of NIPT and of the other test?
   6. What was unappealing?

1. How did you decide whether or not to do NIPT?
   1. Outside of (provider), did you talk about it with any one else?
   2. Did the provider make any recommendations about your situation?
   3. What factors were important when making the decision? [what is emphasized as important]
   4. What information did you use?
   5. Was this a difficult decision to make?
2. {for women who did NIPT} So after deciding you were going to do it, what happened?
   1. What health care providers were involved (e.g. genetic counsellor)?
   2. How long did it take? {did you have to wait for OHIP approval? What was that like}
3. {for women who did NIPT} How did you receive your results?
4. Phone/in person (preference?)
5. What health care providers?
6. How far along were you when you received the results?
7. What about the timing of NIPT results (e.g. how far along you were in pregnancy). Was that ok with you? Do you think you would feel differently if the results showed your baby was affected?
8. Looking back on your experiences with NIPT, what would you recommend to a friend in the same situation?
9. What kind of person would you recommend it to?
10. How would you explain it to them?
11. Do you think it might be helpful for some types of women but not others?
12. If a friend was thinking about doing it, what advice would you give her?
13. What kind of information should women have access to when they are deciding whether or not to do NIPT?
14. How about when they get the results? What type of info should they have access to then?
15. Who should give this info?
16. NIPT has only recently been covered by the health care system, and only for some women. Up until a couple of months ago, women had to pay for it themselves, and it can be quite expensive, $800-$2000. The government is still trying to make an official policy about when they will pay for NIPT. Do you have any thoughts on when, if ever, OHIP should pay for NIPT?
17. If it's not paid for, should it still be offered? For example, anyone could do this test if they are willing to pay for it. Do you think doctors should take time to offer it if you would have to pay on your own? To whom?
18. Should it be offered to every woman, even if they don’t have anything that makes them high risk?
19. When should it be offered?
20. I'm going to ask you a question about whether there should be limits to what type of conditions NIPT should be used to detect, but first I'll give you a bit of information about the scientific possibilities. So, right now, NIPT is only used to test for a small number of conditions, and all of the conditions it is currently used for are also included in other types of prenatal testing, like Down Syndrome. However, it has the possibility to be able to test for many more conditions. Some of them are already possible, like cystic fibrosis or Huntington's disease. Other conditions are still being worked on. Some scientists have succeeded in looking at all of the genes a fetus has using NIPT, which means that in the future they could use NIPT to detect any genetic condition or any trait. This could include conditions that don't start until adulthood, like Huntington's disease. It could detect whether or not a fetus has a higher than average chance of getting breast cancer as an adult. NIPT could be used to detect conditions that aren't disabilities, like whether someone will have hair on the back of their hands or dimples in their cheeks. Does that make sense? I'm just trying to say that right now NIPT only tests for a very small number of conditions compared to what it could be used for, and that it could be used for conditions that most people wouldn't consider to be a disability. Ok, here's the question:
21. Do you think there should be limits on what we can test for prenatally, if we are paying for the test ourselves? {what limits}
22. Do you think there should be limits on what the government will pay for us to test for prenatally?
23. If there should be limits, who should choose what's ok to test for? Who should be involved in this decision? What issues should they consider?
24. How do you distinguish between a disability or disease vs. a trait or condition? What about something like deafness or other conditions that some people would consider a disability but others would just consider a characteristic or a trait, like eye colour or height?
25. What excites you about this technology? What good might it do?
26. What worries you about this technology? What harm might it do?
27. Is there anything else you think we should know about your thoughts or opinions about this technology?
28. Now I just have a few demographic questions before we close: [If they have answered any of these throughout the interview, you don't have to ask again]
29. At the beginning of your pregnancy, was your main pregnancy caregiver a family doctor, midwife, or obstetrician?
30. Before this pregnancy, have you done any other kind of prenatal testing or screening?
31. How old are you?
32. Do you live in a city, town, or rural area?
33. How many children do you have?
34. What's the highest level of education you have?
35. What's your occupation?
36. Do you consider yourself religious? If so, what religion do you belong to?
37. Do you know anyone else who's done this test? (If yes, would you be willing to tell them about our study or pass along a flier?)
38. --- Is it ok if we contact you again if we have any follow up questions? What would be the best way? E-mail? Phone? ----
